# *In Vitro* Cytotoxicity of a Ti-35Nb-7Zr-5Ta Alloy Doped with Different Oxygen Contents

**DOI:** 10.3390/ma7032183

**Published:** 2014-03-13

**Authors:** Tatiani Ayako Goto Donato, Luciano Henrique de Almeida, Victor Elias Arana-Chavez, Carlos Roberto Grandini

**Affiliations:** 1 UNESP—Univ. Estadual Paulista, Laboratório de Anelasticidade e Biomateriais, 17.033-360, Bauru, SP, Brazil; E-Mails: tatianidonato@gmail.com (T.A.G.D.); almeidalh@hotmail.com (L.H.A.); 2 USP—Universidade de São Paulo, Faculdade de Odontologia, Departamento de Biomateriais e Biologia Oral, 05.508-900, São Paulo, SP, Brazil; E-Mail: vearana@usp.br

**Keywords:** Ti-35Nb-7Zr-5Ta alloy, MC3T3-E1 cells, biocompatibility, cell culture

## Abstract

Cp-Ti is the most common material used for dental implants, but its elastic modulus is around five times higher than that of bone. Recently, promising alloys that add Nb, Ta, Zr and Mo to Ti have been developed. The mechanical properties of these alloys are directly related to its microstructure and the presence of interstitial elements, such as oxygen, carbon, nitrogen and hydrogen. In this study, the *in vitro* cytotoxicity of Ti-35Nb-7Zr-5Ta (TNZT) alloys was analyzed in the as-received condition and after being doped with several small quantities of oxygen on a cultured osteogenic cell. The cell’s morphology was also examined by scanning electron microscopy (SEM). The TNZT alloy presented no cytotoxic effects on osteoblastic cells in the studied conditions.

## 1. Introduction

A desirable biomaterial should be predicable and capable of allowing controlled, guided and rapid healing of the host tissue. Cp-Ti is usually considered the material of choice for dental implants, because of its acceptable ductility, corrosion resistance and biocompatibility [1]. However, the elasticity (Young) modulus of Cp-Ti is around five times higher than that of bone, leading to the potential stress of the surrounding residual bone with a detrimental resorptive bone remodeling. A bone, in its natural state, carries its external loads all by itself. When provided with an intramedullary stem, it shares the load-carrying capacity with the implant. Where the same load was first carried by one structure, the bone, it is now carried by two, the stem and the bone. As a consequence, the bone is subjected to reduced stresses, hence being stress shielded. Then, stress shielding is a mechanical phenomenon, occurring in composites of stiff and flexible materials [2].

Several titanium-based alloys have been used as biomaterials for implantology; among these, the Ti-6Al-4V (TAV) alloy is the most commonly employed. Although the inclusion of aluminum and vanadium improves the elastic modulus of titanium, recent studies have shown that these elements could cause toxic effects on tissues. In addition, a high amount of aluminum interferes with cell viability, besides having a long-term cytotoxic effect *in vitro* and *in vivo* [3,4]. Moreover, this alloy still has a relatively high modulus (about 120 GPa) when compared with that of bone (about 28 GPa) [5].

In spite of reducing the elasticity modulus of titanium alloys without causing cytotoxic effects, new promising alloys that add niobium, tantalum, zirconium and molybdenum to titanium have been developed. These alloys represent a new class of titanium-based alloys, which are free of aluminum and vanadium, while exhibiting low values of the Young’s modulus; these qualities render them quite attractive as biomaterials [5,6]. The Ti-35Nb-7Zr-5Ta (TNZT) alloy is, among the titanium-based alloys, the one that presents the smallest value of the elasticity modulus [7]. It is a beta-type alloy that lacks toxic elements, such as aluminum and vanadium. *In vitro*, aluminum accelerated osteoblastic differentiation and nodule formation, but was cytotoxic in the long term [8].

The mechanical properties of titanium-based alloys are directly related to their microstructure [9,10]. In fact, the strength and toughness of many β alloys are improved by the presence of the ω phase. The reason behind such a coexistence was attributed to the fact that α forms in a compositionally invariant way, having no long-range effect on the β matrix [11]. β alloys usually have good response to heat treatment, weldability, ductility and toughness, but poor creep-resistance. This spontaneous tendency to form grain boundary α along with the low β transus makes it difficult to achieve high toughness in these alloys [12]. Indeed, the presence of interstitial elements (such as oxygen, carbon, nitrogen and hydrogen) causes strong changes in the mechanical properties of metals, mainly in their elastic properties, by hardening or softening the alloy [13–15]. Young’s modulus is well-known to depend on the nature of atomic bonding, but is independent of the microstructure of the materials, because the bonding nature originates from the atomic configuration of each component of the material [16].

Though this alloy has been reported to have superior mechanical properties, the biocompatibility is relatively not known. A previous *in vitro* study showed that the TNZT alloy in its as-received state has no direct or indirect cytotoxic effect on fibroblasts [17], which indicates that it is a promising alloy for use as a biomaterial. However, studies involving bone cells, namely osteoblasts, should be performed to support this idea.

The aim of the present study is to investigate the biocompatibility of a TNZT alloy doped with different oxygen contents on bone cells by using *in vitro* cytotoxicity tests and scanning electron microscopy.

## 2. Results and Discussion

Table 1 shows the chemical analysis of the prepared samples. The results show that the concentration of substitutional elements is very close to the stoichiometry, demonstrating the good quality of the produced samples. The obtained values for the oxygen content in the samples are presented in Table 2.

**Figure 1.**
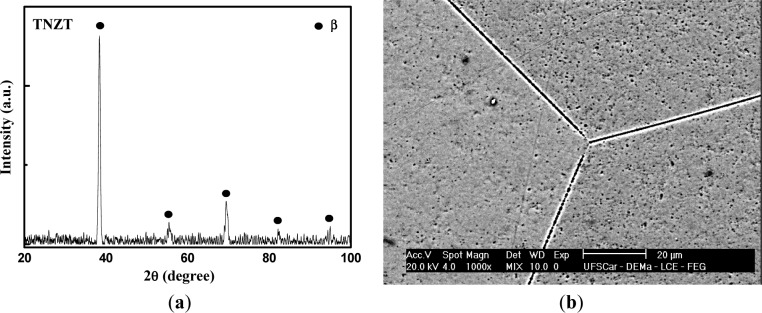
(**a**) XRD patterns; and (**b**) SEM microstructures of the TNZT alloy after homogenization heat treatment for 6 h at 1273 K, followed by furnace cooling.

Part (a) of Figure 1 shows the X-ray diffractogram for the TNZT sample after homogenization heat treatment. The presence of the peaks relating to β the phase of the alloy can be observed, showing a good stability and the predominance of this phase in the samples. The SEM micrograph of the TNZT sample is presented in part (b) of Figure 1. A microstructure comprising grains characteristic of heat treated material, with morphology typical of the β phase, can be observed in the micrograph.

**Figure 2.**
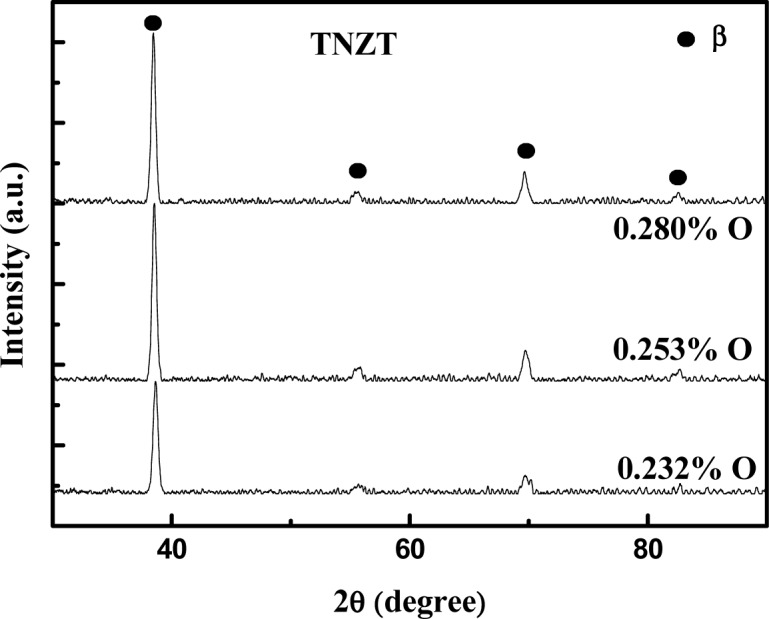
XRD patterns for the TNZT alloy with 0.232%, 0.253% and 0.280% of oxygen.

**Figure 3.**
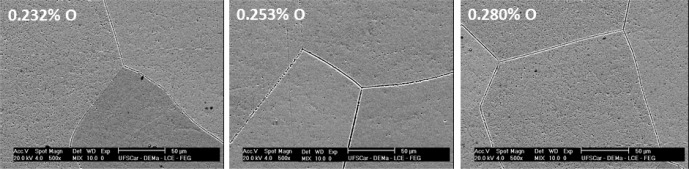
SEM microstructures for the TNZT alloy with 0.232%, 0.253% and 0.280% of oxygen.

The effect of oxygen concentration on the structure was analyzed by XRD. Figure 2 shows the XRD pattern of the samples with 0.232% O, 0.253% O and 0.280% O. Only the peaks from the β phase were detected. These results indicate that the increase in the oxygen concentration was not sufficient for the formation of the α’ martensite phase. The micrographs of the alloys after the doping steps are shown in Figure 3 and show a microstructure typical of the β phase, too. The variation in the interstitial oxygen concentration did not cause changes in the microstructure, as shown earlier by the results of X-ray diffraction.

**Figure 4.**
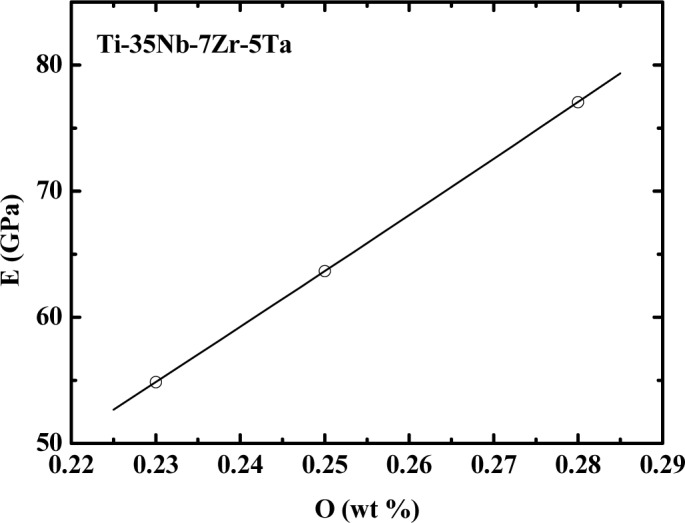
The variation of the elasticity modulus of the TNZT samples as a function of the oxygen concentration.

Figure 4 shows the variation of the modulus as a function of oxygen concentration for all studied samples, measured at room temperature. An increase in the modulus of elasticity can be observed when the oxygen concentration is increased. This effect is associated with the arrangement of the interstitial oxygen atoms within the β phase, which restricts the movement of linear defects [6,18]. The increased oxygen concentration can result in α-phase precipitation (which was not observed in the DRX or SEM measurements), contributing to the increase in the modulus of elasticity [6,18].

**Figure 5.**
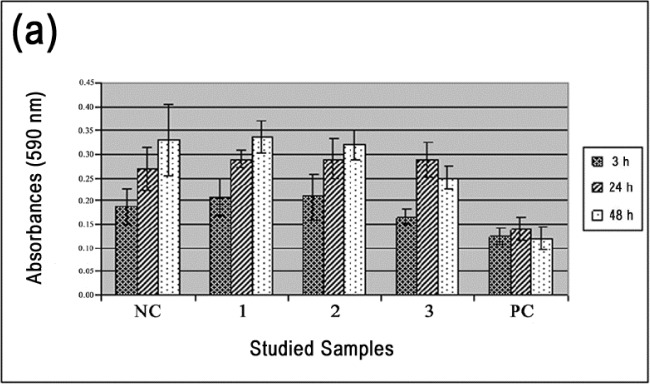

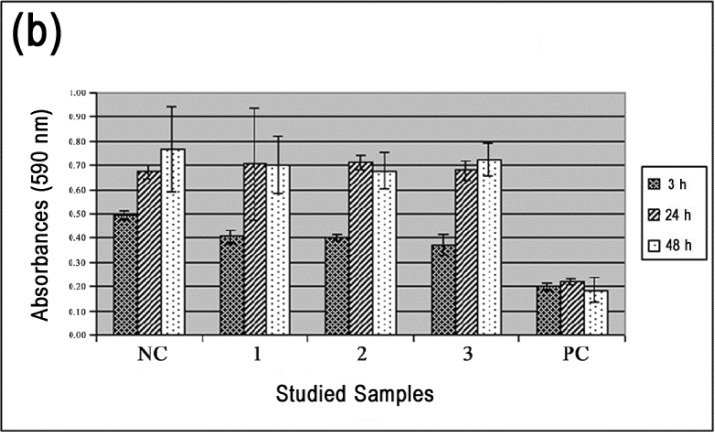
Evolution of the cytotoxicity of TNZT alloy doped with three amounts of oxygen. (**a**) Direct and (**b**) indirect MTT (3-(4,5-dimethyl-2-thiazolyl)-2,5-diphenayl-2H-tetrazolium bromide); NC (negative control); (1) the TNZT #1 sample; (2) the TNZT #2 sample; and (3) the TNZT #3 sample and PC (positive control).

The MC3T3-E1 cells were well adhered to all the materials tested in the first time period (3 h). Figure 5 shows the absorbance measurements for the TNZT alloys studied for both direct and indirect cytotoxical MTT tests after the three time periods (3, 24 and 48 h).

Figure 5a shows the results of the direct cytotoxic test for the TNZT samples in all studied conditions. The number of viable cells cultured on polystyrene, TNZT #1 and TNZT #2 increased with time, but slightly decreased for TNZT #3. However, TNZT #3 was higher than the positive control in the last time period (48 h). The differences were not statistically significant (*p* = 0.05). In general, the viable cells for the positive control were significantly less than those of all the other materials tested.

Figure 5b shows the results of the indirect cytotoxic test for the TNZT samples in all studied conditions. The number of viable cells cultured on all the materials was similar. They increased from the first to the second time period, but remained almost without variation in the third period. Thus, no statistically significant differences occurred (*p* = 0.05). As for the direct cytotoxicity test, the viable cells for the positive control were significantly less than those of all the other materials tested.

**Figure 6.**
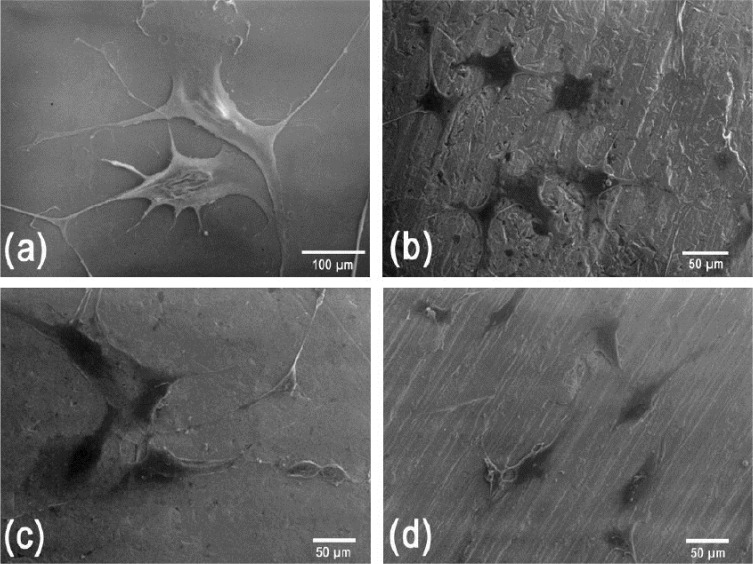
Scanning electron micrographs of cells grown on TNZT alloy samples doped with three amounts of oxygen. (**a**) Control samples: glass; (**b**) the TNZT #1 sample; (**c**) the TNZT #2 sample; (**d**) the TNZT #3 sample. The predominant cell shape was polygonal, with cells exhibiting a central and flattened cell body and numerous and long processes.

Figure 6 shows the SEM analysis for the TNZT samples in all the studied conditions. All the studied materials showed cells with a similar morphology. The cells were observed to be well-adhered to the TNZT #1, 2 and 3 samples, as well as to the glass plate. All of them, independent of the material, showed a central and flattened cell body, as well as numerous and long processes.

Previous findings that showed the biocompatibility of the non-doped TNZT alloy with cultured fibroblasts [17] were confirmed by employing bone cells. MC3T3-E1 cells are a preosteoblastic immortalized lineage obtained from mouse calvaria that are largely used in numerous *in vitro* studies [19]. The cells exhibited solid adhesion to all the tested alloys, as well as to polystyrene in the cytotoxic analyses and to glass in the micromorphological evaluation. In all cases, the absorbance levels that determined the presence of viable cells were similar; the few variations were not statistically significant.

This study showed that the introduction of oxygen did not affect cell viability, as can be seen in the MTT direct and indirect tests, in which none of the treatments showed cytotoxic effects. The cell adhesion was analyzed by scanning electron microscopy, which allowed for the verification of positive cell interaction with the TNZT alloys in all of the studied conditions. Cell morphology was preserved, showing no kind of aggression caused by the material under study.

The advantage of this biomaterial, when compared to commercial alloys, is the absence of cytotoxic elements, such as Al and V. On the other hand, the introduction of oxygen into a solid solution showed an increase in the elasticity modulus. The TNZT alloy presents several attractive features, such as the nearness of its elastic modulus to the bone [5,20], in addition to corrosion and mechanical resistance and biocompatibility, as previously seen by Donato *et al.* [17].

## 3. Experimental Section

In this study, TNZT alloy samples were prepared in an arc-melting furnace with a cooled crucible. Ti, Nb, Zr and Ta, with a purity of 99.99% (Sigma-Aldrich Inc., St. Louis, MI, USA), were used. After melting, the alloy ingots were swaged to take the form of rods with an approximately 3.0-mm diameter. The chemical analysis of the samples after melting was made using a Vista plasma atomic emission equipment (Varian Inc., Cary, NC, USA). The analyses were made in five different regions of the samples. The prepared samples were submitted to an annealing in an ultra-high vacuum (5 × 10^−9^ Torr) at 1273 K for 6 h, followed by furnace cooling, for internal stress relief from the processing of samples.

The structure of the samples was obtained by X-ray diffraction (XRD) measurements in a D/Max 2100/PC diffractometer (Rigaku, Tokyo, Japan) using the powder method with Cu-Kα (λ = 1.544 Ǻ) radiation in continuous time mode with a scan speed of 2°/min in the range of 3° to 100°. A microstructural analysis was performed using a XL-30 FEG microscope (Philips, New York, NY, USA).

To vary the amount of interstitial elements, the samples were doped with oxygen. After the annealing, the samples were heated for two hours in an oxygen atmosphere at a temperature of 1073 K and partial pressures of 1.0 × 10^−5^ (TNZT #1), 5.0 × 10^−4^ (TNZT #2) and 1.0 × 10^−4^ (TNZT #3) Torr and were fast-cooled to room temperature. The interstitial contents were obtained using a TC-136 apparatus (Leco Co., St. Joseph, PA, USA). The method used was melting the samples under inert gas, with infrared detection for oxygen and thermal conductivity differences for nitrogen. For each sample, the measurements were made in five different regions of the sample [21].

The elasticity modulus was obtained in a torsion pendulum, with an oscillation frequency of 30 Hz, under a vacuum of 10^−6^ Torr with a heating rate of 1 K/min and a temperature range of 250 to 350 K [22,23].

TNZT discs (~4 mm in diameter and 2 mm-thick) were cleaned by sonication and then autoclaved.

MC3T3-E1 cells are a preosteoblastic lineage, obtained from the calvaria of *Mus musculus* (ATCC, Rockville, MD, USA). Cells were grown in a complete alpha-minimum essential medium (α-MEM, Invitrogen, Carlsbad, CA, USA) and supplemented with 10% fetal bovine serum (FBS) and 1% gentamicin (Invitrogen). The cells were incubated at 37 °C in a humidified atmosphere with 5% CO_2_. The progression of cultures was examined by phase-contrast microscopy of cells grown on polystyrene. The medium was changed every 2–3 days [19].

A standard colorimetric assay MTT [3-(4,5-dimethyl-2-thiazolyl)-2,5-diphenayl-2H-tetrazolium bromide] has been used to demonstrate the cell viability and proliferation, as well as the cytotoxicity based on reduction of the MTT. This reduction takes place only when mitochondrial reductase enzymes are active; thus, conversion is directly related to the number of viable cells [24–26]. In direct cytotoxicity, MC3T3-E1 cells (with a density of 2 × 10^4^ cell/well) were plated under the surface of the TNZT discs that were placed on 96-well microplates. In the indirect cytotoxicity, the extracts of the alloys were obtained, and then, the culture medium was substituted by the obtained extracts. In both tests, following incubation for 3, 24 and 48 h, MTT (5mg/mL; Sigma Co., St Louis, MO, USA) was added to each well, and the plate was incubated at 37 °C for 3 h. After this period, the medium was removed, and 100 μL DMSO (dimethyl sulfoxide) were added to dissolve the formazan crystals. The product was quantified in the spectrophotometer by measuring absorbance at 562 nm using a SpectraMax Plus microplate reader (Molecular devices, Sunnyvale, CA, USA). The polystyrene extract was used as the negative control, while a solution of α-MEM, 10% of FBS and 1% of phenol was the positive control.

The qualitative analysis was made using scanning electron microscopy (SEM) to observe cell morphology when plated in direct contact with the TNZT alloy. The MC3T3-E1 cells were plated (with a density of 2 × 10^4^ cell/well) under the surface of the alloys. After 24 h, the cells were fixed in 4% paraformaldehyde + 0.1% glutaraldehyde buffered with sodium cacodylate, pH 7.2, for 1 h at room temperature and were then routinely processed for SEM. Samples were examined post-fixed with 1% osmium tetroxide, dehydrated in crescent concentrations of ethanol and desiccated after treatment with HMDS. Specimens were mounted onto aluminum substrates, sputtered with gold, and examined in a LEO 430 scanning electron microscopy. Cells grown on a glass coverslip were used as a negative control [27].

Results were analyzed statistically by an analysis of variance between groups **(**ANOVA**)** test complemented with a Tukey’s test. The level of significance was 5% (*p* ≤ 0.05).

## 4. Conclusions

In this paper, biocompatibility studies were performed with Ti-35Nb-7Zr-5Ta alloys that contained several quantities of oxygen in a solid solution.

The gas doping process was effective for the insertion of interstitial oxygen in the alloys. From the results of X-ray diffraction and scanning electron microscopy, it was possible to observe that the oxygen concentrations did not cause significant changes in the crystalline structure and microstructure of the alloys.

In direct or indirect cytotoxicity tests, none of the studied alloys presented a toxic effect for the cells. The Ti alloys tested presented a good *in vitro* biocompatibility.

The presence of oxygen, in turn, did not interfere with the *in vitro* biocompatibility results. Thus, oxygen in a solid solution can be used in an attempt to improve the mechanical properties of the Ti alloys studied.

In general, the Ti alloys studied did not present any cytotoxicity, whether directly or indirectly. On the contrary, the SEM results presented illustrations of cellular division by perfectly maintaining the morphology, thus indicating good integration between the cells and the materials.

## Figures and Tables

**Table 1.** Chemical analysis of the Ti-35Nb-7Zr-5Ta (TNZT) alloys.

**Element**	Ti	Nb	Zr	Ta
**Content (wt%)**	balance	(35.2 ± 0.4)	(7.5 ± 0.4)	(5.0 ± 0.2)

**Table 2.** Oxygen concentration in the TNZT samples.

**Sample**	TNZT #1	TNZT #2	TNZT #3
**O content (wt%)**	(0.232 ± 0.004)	(0.253 ± 0.006)	(0.28 ± 0.03)

## References

[1] Steinemann S.G. (1998). Titanium—The material of choice?. Periodontology 2000.

[2] Huiskes R., Weinans H., Rietberg B.V. (1992). The relationship between stress shielding and bone resorption around total hip stems and the effects of flexible materials. Clin. Orthop. Relat. Res.

[3] Brunette D.M., Tengvall P., Textor M., Thomsen P. (2001). Titanium in Medicine: Material Science, Surface Science, Engineering, Biological Responses and Medical Applications.

[4] Li Y., Wong C., Xiong J., Hodgson P., Wen C. (2010). Cytotoxicity of titanium and titanium alloying elements. J. Dent. Res.

[5] Geetha M., Singh A.K., Asokamani R., Gogia A.K. (2009). Ti based biomaterials, the ultimate choice for orthopaedic implants—A review. Prog. Mater.Sci.

[6] Rack H.J., Qazi J.I. (2006). Titanium alloys for biomedical applications. Mater. Sci. Eng. C.

[7] Niinomi M., Nakai M. (2011). Titanium-based biomaterials for preventing stress shielding between implant devices and bone. Int. J. Biomater.

[8] Bellows C.G., Heersche J.N.M., Aubin J.E. (1999). Aluminum accelerates osteoblastic differentiation but is cytotoxic in long-term rat calvaria cell cultures. Calcif. Tissue Int.

[9] Ankem S., Greene C.A. (1999). Recent developments in microstructure/property relationships of beta titanium alloys. Mater. Sci. Eng. A.

[10] Wang L., Lu W., Qin J., Zhang F., Zhang D. (2008). Microstructure and mechanical properties of cold-rolled tinbtazr biomedical β titanium alloy. Mater. Sci. Eng. A.

[11] Azimzadeh S., Rack H.J. (1998). Phase transformations in Ti-6.8Mo-4.5Fe-1.5Al. Metall. Mater. Trans. A.

[12] Trinkle D.R., Hennig R.G., Srinivasan S.G., Hatch D.M., Jones M.D., Stokes H.T., Albers R.C., Wilkins J.W. (2003). A new mechanism for the alpha to omega martensitic transformation in pure titanium. Phys. Rev. Lett.

[13] Besse M., Castany P., Gloriant T. (2011). Mechanisms of deformation in gum metal TNTZ-O and TNTZ titanium alloys: A comparative study on the oxygen influence. Acta Mater.

[14] Duan H.-P., Xu H.-X., Su W.-H., Ke Y.-B., Liu Z.-Q., Song H.-H. (2012). Effect of oxygen on the microstructure and mechanical properties of Ti-23Nb-0.7Ta-2Zr alloy. Int. J. Miner. Metall. Mater.

[15] He W.J., Zhang S.H., Song H.W., Cheng M. (2009). Hydrogen-induced hardening and softening of a β-titanium alloy. Scr. Mater.

[16] Tane M., Nakano T., Kuramoto S., Hara M., Niinomi M., Takesue N., Yano T., Nakajima H. (2011). Low young’s modulus in Ti–Nb–Ta–Zr–O alloys: Cold working and oxygen effects. Acta Mater.

[17] Donato T.A.G., de Almeida L.H., Nogueira R.A., Niemeyer T.C., Grandini C.R., Caram R., Schneider S.G., Santos A.R. (2009). Cytotoxicity study of some ti alloys used as biomaterial. Mater. Sci. Eng. C.

[18] Qazi J.I., Marquardt B., Allard L.F., Rack H.J. (2005). Phase transformations in Ti-35Nb-7Zr-5Ta-(0.06–068)O alloys. Mater. Sci. Eng. C.

[19] International Organization for Standardization (ISO) (2009). Biological Evaluation of Medical Devices—Part 5: Tests for in Vitro Cytotoxicity.

[20] Niinomi M. (2008). Biologically and mechanically biocompatible titanium alloys. Mater. Trans.

[21] Almeida L.H., Grandini C.R., Caram R. (2009). Anelastic spectroscopy in a Ti alloy used as biomaterial. Mater. Sci. Eng. A.

[22] Grandini C.R. (2002). A low cost automatic system for anelastic relaxations measurements. Rev. Bras. Apli. Vácuo.

[23] Pintão C.A.F., Almeida L.H.D., Grandini C.R. (2006). Medida do momento de inércia de um pêndulo de torção para estudo de relaxações anelásticas. Rev. Bras. Apli. Vácuo.

[24] Mosmann T. (1983). Rapid colorimetric assay for cellular growth and survival: Application to proliferation and cytotoxicity assays. J. Immunol. Methods.

[25] Arisu H., Türköz E., Bala O. (2006). Effects of Nd:Yag laser irradiation on osteoblast cell cultures. Lasers Med. Sci.

[26] Berridge M.V., Tan A.S. (1993). Characterization of the cellular reduction of 3-(4,5-dimethylthiazol-2-yl)-2,5-diphenyltetrazolium bromide (MTT): Subcellular localization, substrate dependence, and involvement of mitochondrial electron transport in mtt reduction. Arch. Biochem. Biophys.

[27] Nanci A., Zalzal S., Gotoh Y., McKee M.D. (1996). Ultrastructural characterization and immunolocalization of osteopontin in rat calvarial osteoblast primary cultures. Microsc. Res. Tech.

